# Diploic Bone Channel Drilling Facilitates Dissection of the Midline Dura and Protects the Superior Sagittal Sinus in Hyperostosis Frontalis Interna

**DOI:** 10.7759/cureus.35704

**Published:** 2023-03-02

**Authors:** Martin Rutkowski, Ahmad Ozair, Brian Niehaus, Michael W McDermott

**Affiliations:** 1 Department of Neurosurgery/Department of Otolaryngology, Medical College of Georgia, Augusta University, Augusta, USA; 2 Department of Neurosurgery, University of California, San Francisco, San Francisco, USA; 3 Miami Cancer Institute, Baptist Health South Florida, Miami, USA; 4 Miami Neuroscience Institute, Baptist Health South Florida, Miami, USA; 5 Division of Neuroscience, Herbert Wertheim College of Medicine, Florida International University, Miami, USA

**Keywords:** dural-based tumor, hyperostosis frontalis interna, inner table, meningioma, bone flap, craniectomy, craniotomy, diploic bone, hyperostosis, superior sagittal sinus

## Abstract

Patients with space-occupying lesions adjacent to the superior sagittal sinus (SSS) present several technical considerations. For craniotomies crossing the SSS, a two-part method allows for dissection of the epidural space and dura under direct vision after removing a more lateral parasagittal bone flap. However, when the inner table surface of the medial component of the two-part bone flap is irregular, this can be difficult. We describe a method for channel drilling of the diploic bone, which allows for the piecemeal removal of the inner table using an upbiting rongeur.

This article presents the case of meningioma with documented growth and provides a technical note of this technique to facilitate safe dissection of the midline dura. A patient presented with headaches and an anterior one-third parasagittal meningioma with documented growth. She selected surgical removal for treatment. A right frontal two-part parasagittal craniotomy was recommended. The preoperative imaging showed that the frontal bone was thick, with irregularity of the inner table. Intraoperatively, a channel was drilled in the diploic space of the bone, leaving the outer table intact. This provided a thin lip of the inner table that could be dissected over a short distance and then removed with a 2-mm upbiting rongeur. This allowed for further dissection of the dura crossing the midline under direct vision and safe secondary bone piece removal. The dura was opened to the edge of the SSS, allowing full exposure of the parasagittal region and interhemispheric fissure, thus limiting retraction of the medial right frontal lobe. The bone flap was removed in two pieces without a dural tear over the midline in spite of inner table irregularities. A Simpson grade 1 removal was accomplished, including excision of the affected falx, and the postoperative course was uncomplicated. In conclusion, diploic bone channel drilling is a technique that can be used to create a thin lip of the inner table, which can be removed piecemeal for safe dissection of the midline dura crossing the midline.

## Introduction

Meningiomas arising in the falx cerebri and from the parasagittal region together constitute one of the most common locations of intracranial meningiomas. Surgical removal of these falcine and parasagittal meningiomas, particularly during a unilateral approach, requires adequate exposure of the midline to avoid undue retraction of the medial ipsilateral cerebral hemisphere. Key technical considerations include avoidance of injury to the superior sagittal sinus (SSS) and management of frontal sinus transgression when approaching very anterior extra-axial masses.

Further technical challenges to the safe resection of tumors in the anterior one-third region are presented by thick bone and/or irregularities of the inner table related to tumor-associated hyperostosis or hyperostosis frontalis interna (HFI). Tumor-associated hyperostosis is usually restricted to a small area immediately over the tumor, while the latter condition, HFI, can affect the inner table of the entire frontal region. HFI is a condition defined by bone deposition and thickening, usually limited to the inner table of the frontal bone, often sparing the midline. It has a prevalence of 5%-12%, more often affects women than men, and appears to be associated with elderly postmenopausal women in particular, with an incidence of 40%-60% [1]. Histopathological investigation of HFI reveals a widened zone of lamellar bone and possible endocranial plate remodeling [2].

Extra-axial pathology, including meningiomas, frequently warrants an increased exposure of the sagittal sinus and the falx cerebri to facilitate more relaxed retraction of the frontal lobe and improve the safety and manipulation of the tumor resection. Achieving this exposure necessitates craniotomy and frontal bone removal over the SSS; we have used what we call a “two-part parasagittal craniotomy” to help with the safe dissection of the midline dura crossing the SSS to the opposite side [3].

We describe a case, and associated surgical technique, of right frontal parasagittal meningioma with both thick frontal bone and an irregular inner table where a channel drilling of the diploic bone was done to provide a thin edge of the inner table, which could be dissected over short distances and then removed piecemeal. This allowed the safe dissection of the dura over the midline and the creation of a small secondary bone flap giving full exposure of the midline.

## Technical report

Case presentation

**Figure 1:**
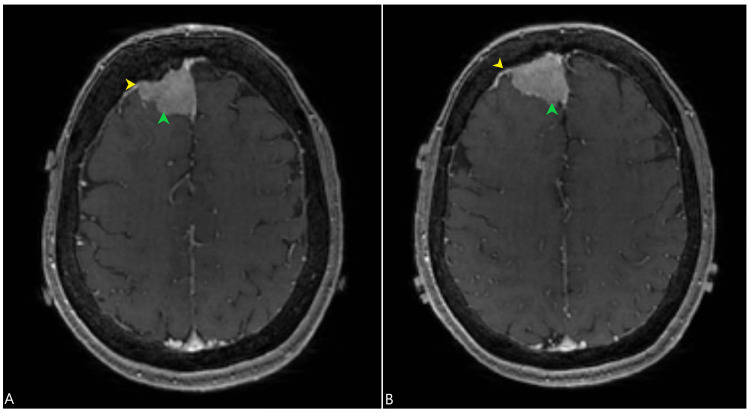
Post-contrast (gadolinium-based) axial MR imaging of the brain showing a right frontal parasagittal meningioma (green arrowheads) along with hyperostosis of the right frontal bone (yellow arrowheads) shown in two different MR sections (A and B). MR: magnetic resonance

A 64-year-old female was referred to the Department of Neurosurgery of the University of California, San Francisco, with new-onset headaches. She underwent magnetic resonance (MR) imaging, which demonstrated a right frontal parasagittal meningioma with bony irregularities of the inner table of the skull, consistent with hyperostosis frontalis interna (Figure 1).

**Figure 2:**
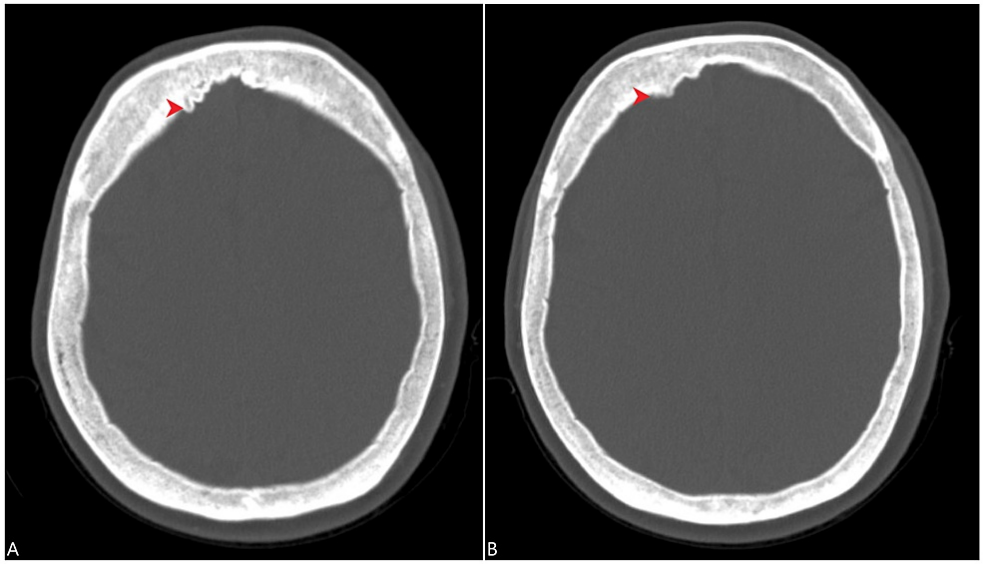
CT scan of the head demonstrated hyperostosis of the right frontal bone (red arrowheads) in the bone window, as shown here in two different sections (A and B). CT: computed tomography

Computed tomography (CT) imaging also demonstrated hyperostosis of the inner table of the skull (Figure 2). Interval imaging revealed more than 2 mm of growth in six months, and the options of further observation, radiotherapy, radiosurgery, and microsurgery were discussed with the patient. After a shared decision-making process, the patient elected to pursue surgical removal of the meningioma.

Surgical anatomy

Relevant anatomy related to this technique is reviewed below. Following the exposure of the frontal bones, the sagittal suture can be appreciated as an irregular grooved diastatic suture that extends from anterior to posterior over the midline of the skull. The sagittal suture is intersected by the coronal suture that defines the posterior border of the frontal bones, along with the anterior border of the parietal bones, being approximately 4-5 cm in front of the precentral gyrus. Underlying this suture is the superior sagittal sinus (SSS), a venous channel contained within the periosteal and meningeal layers of the dura that drains venous blood anterior to posterior. The SSS receives a large and variable number of tributaries from the adjacent superficial cerebral cortex, with accidental damage and bleeding from this structure being challenging to control. It is proposed that the redundancy and collateral venous flow into the SSS renders the anterior one-third of the SSS safely resectable. However, avoidance of injury wherever possible remains a core surgical tenet.

Operative technique

**Figure 3:**
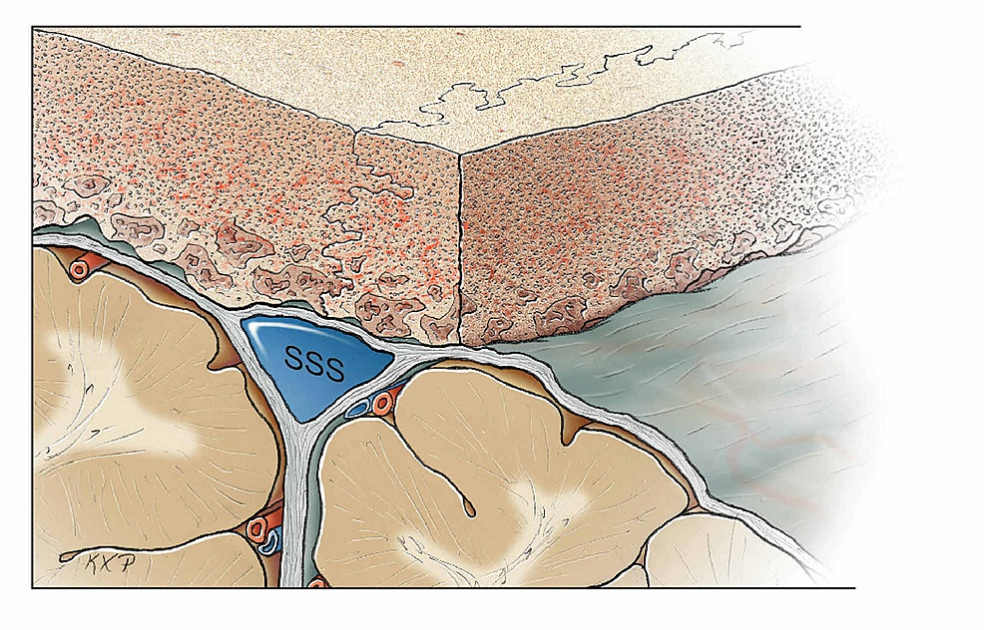
Parasagittal bone flap removed as a first step. SSS: superior sagittal sinus Original figure by Ken Probst, University of California, San Francisco

At surgery, a coronal incision was fashioned. The pericranial layer was preserved and incised medial to the superior temporal line bilaterally and then dissected and reflected forward. Image guidance was used to plan a two-part parasagittal bone flap crossing the midline. The initial lateral bone flap had to be removed with a longer footplate and cutting blade attachment due to the frontal bone thickness, just lateral to the SSS (Figure 3).

**Figure 4:**
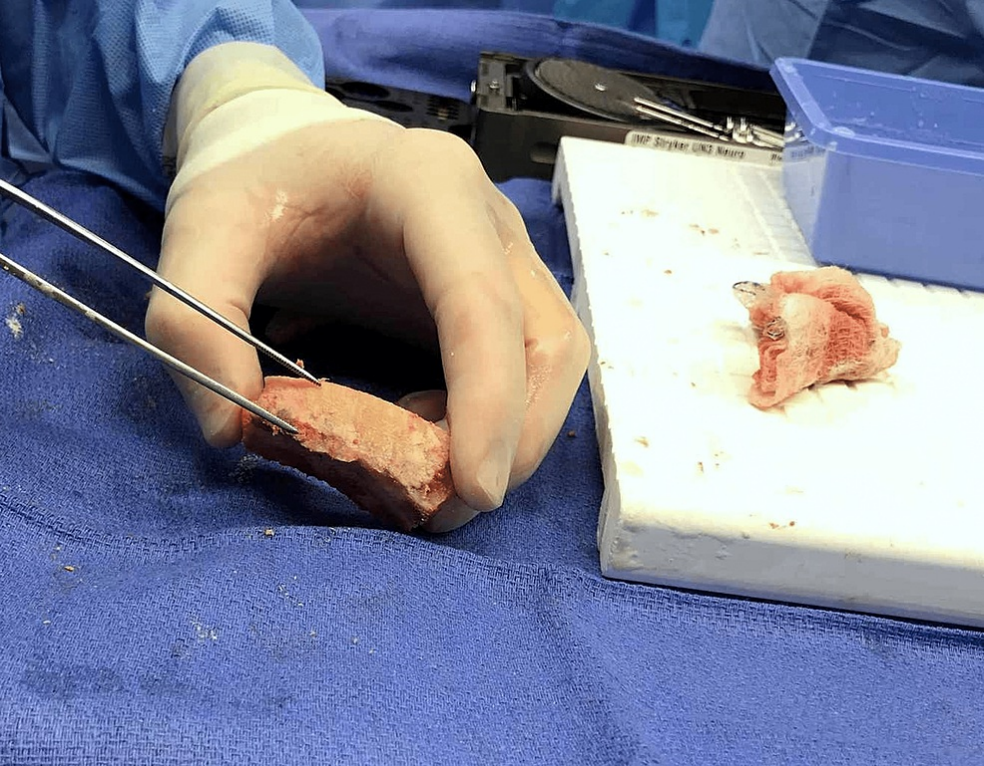
Intraoperative image demonstrating the thickening and irregularity of the inner table of the frontal bone, indicating “hyperostosis frontalis interna.”

**Figure 5:**
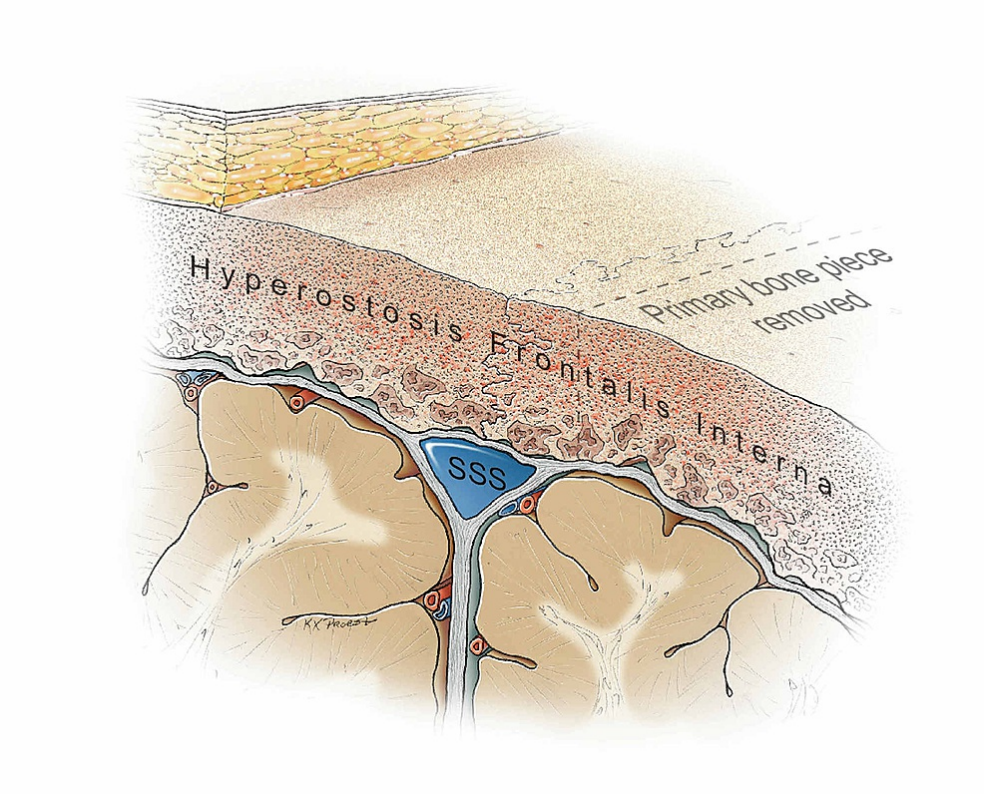
Irregularity of the inner table of the frontal bone adjacent to the superior sagittal sinus SSS: superior sagittal sinus Original figure by Ken Probst, University of California, San Francisco

Once the lateral bone flap was off, an inspection of the inner table in the parasagittal region revealed it to be irregular over the length of the exposure (Figure 4 and Figure 5).

**Figure 6:**
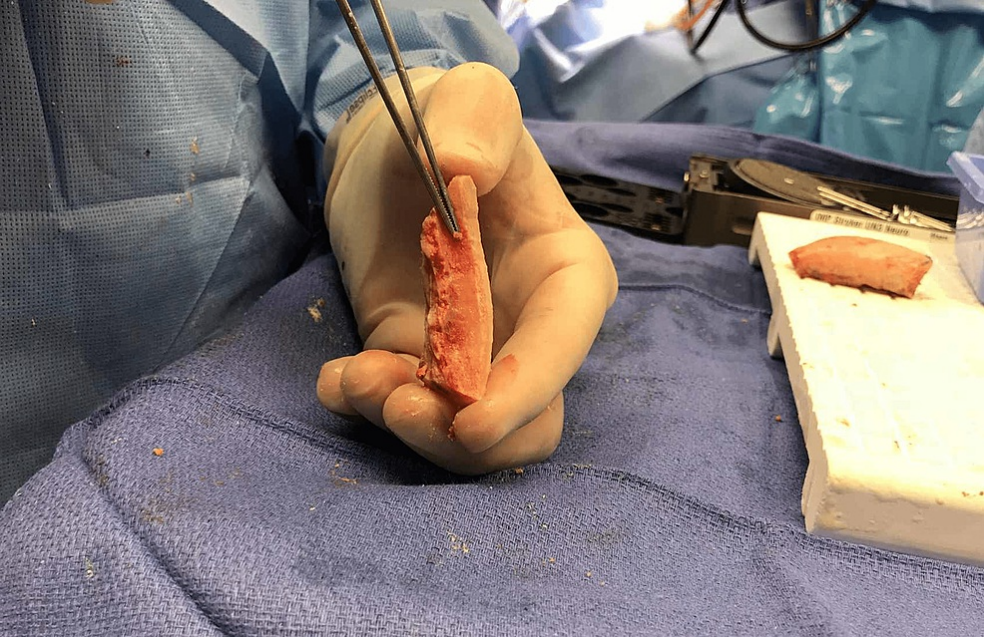
Intraoperative image showing drilling of diploic space to facilitate dissection

**Figure 7:**
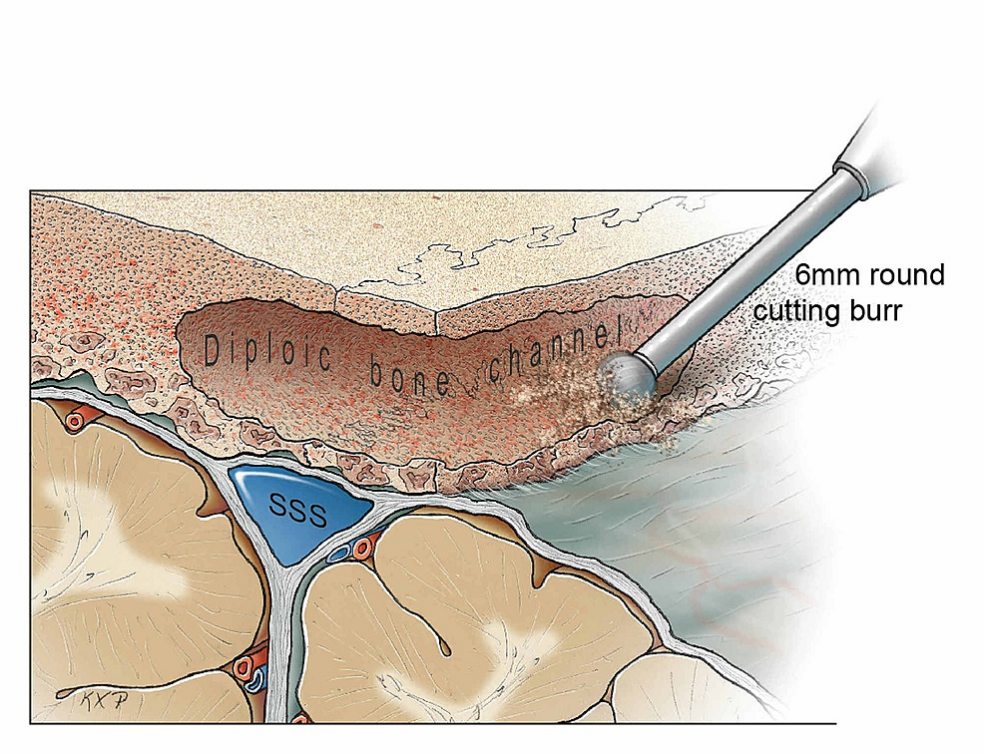
Drilling of the diploic channel to create a thin rim that can be carefully removed with an upbiting Kerrison rongeur. SSS: superior sagittal sinus Original figure by Ken Probst, University of California, San Francisco

Initial attempts to dissect the dura with a Penfield 1 dissector were unsuccessful. Therefore, a 6-mm round cutting burr, the same instrument used to create the anterior and posterior parasagittal burr holes, was used to drill a channel in the diploic bone (Figure 6 and Figure 7).

**Figure 8:**
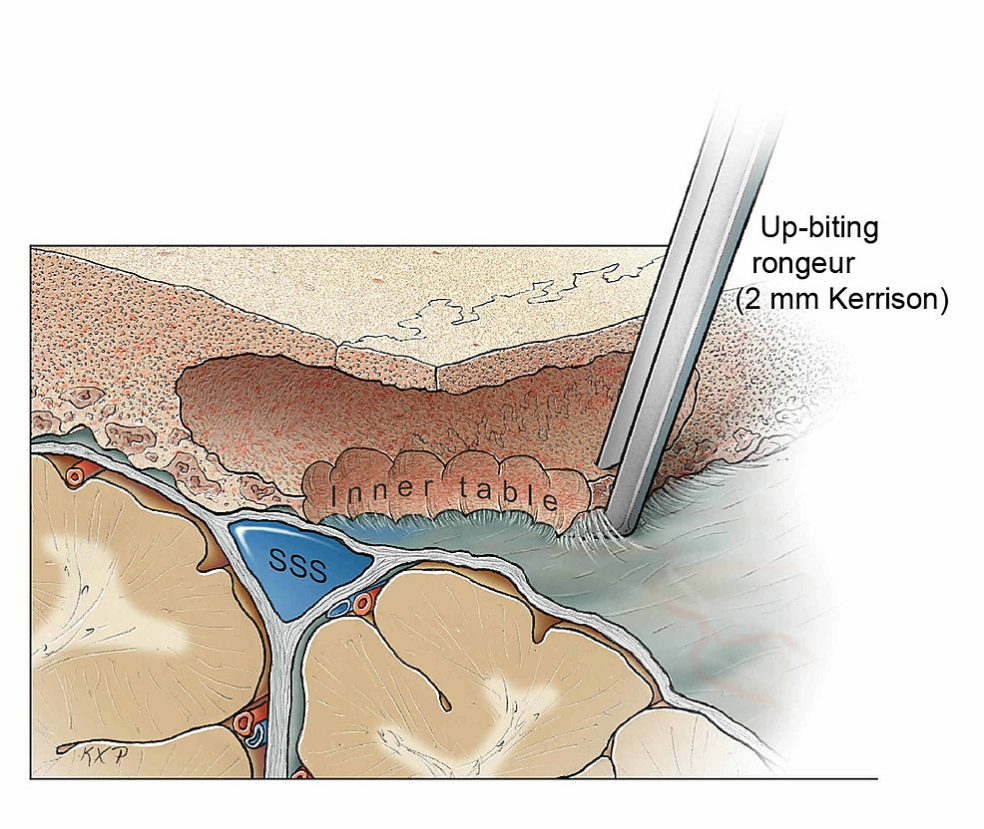
Drilling of the diploic channel to create a thin rim of the inner table that can be safely removed with an upbiting Kerrison rongeur. SSS: superior sagittal sinus Original figure by Ken Probst, University of California, San Francisco

**Figure 9:**
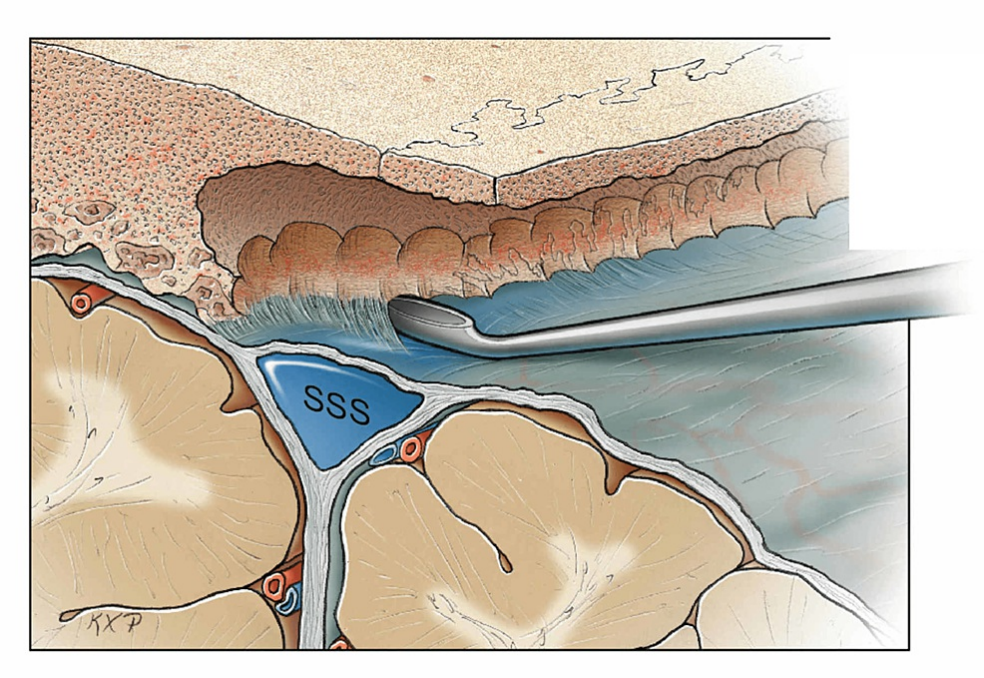
Safe dissection across the midline using a periosteal dissector. A Penfield 1 may also be utilized. SSS: superior sagittal sinus Original figure by Ken Probst, University of California, San Francisco

The drilling of diploic bones permitted the creation of a thin lip of the inner table (Figure 8 and Figure 9). This thin lip was removed with a 2-mm upbiting rongeur in a piecemeal fashion over the length of the medial bone edge so that less irregular bone was exposed, allowing safe dissection of the midline. Ensuring that the outer table remained intact was a key consideration in preventing a bony defect across the midline during reconstruction.

**Figure 10:**
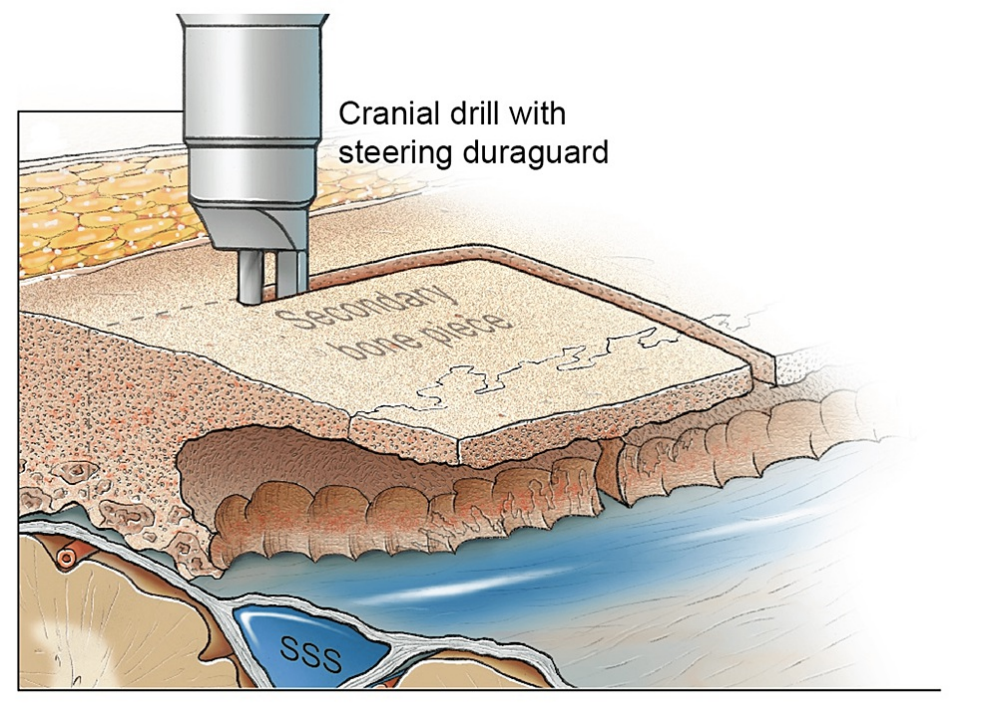
Safe removal of the secondary bone piece across the midline providing full exposure to the midline. Original figure by Ken Probst, University of California, San Francisco

The secondary medial bone piece crossing the midline was then removed without incident (Figure 10). The SSS was noted to be intact following the craniectomy. Hemostasis on the midline was obtained with the bipolar cautery forceps and a 1” × 3” piece of Gelfoam (Pfizer Inc., New York, USA).

Results

**Figure 11:**
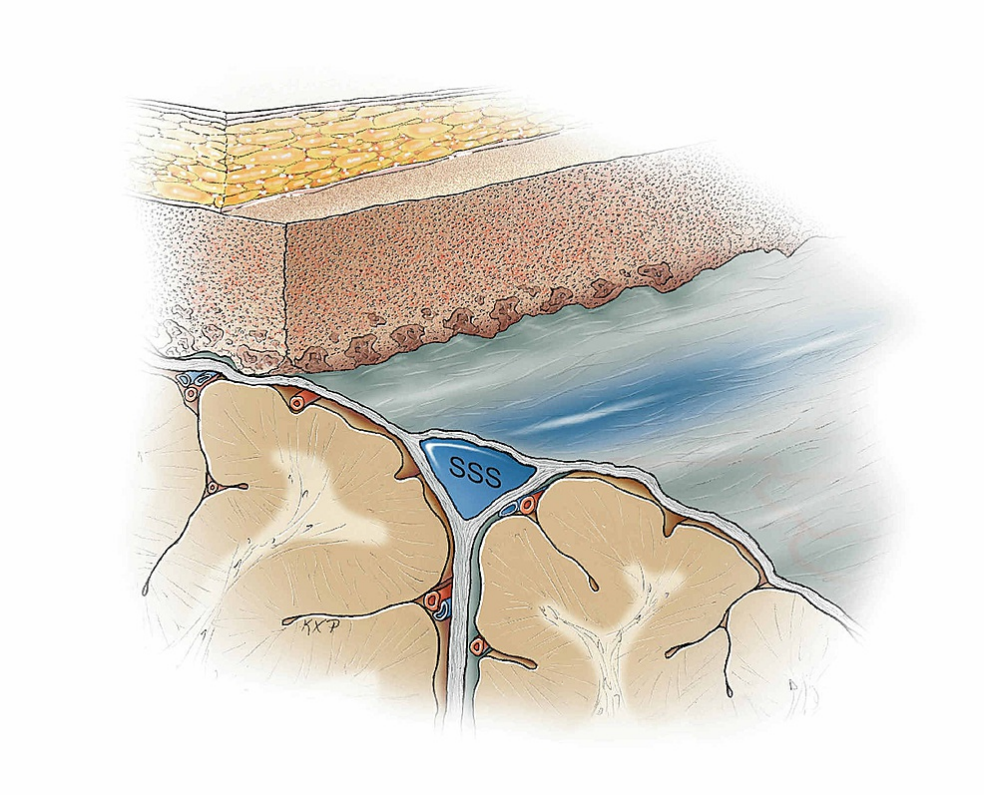
Final result of the two-part parasagittal craniotomy technique with diploic bone drilling to avoid injury to the superior sagittal sinus. Original figure by Ken Probst, University of California, San Francisco

Utilization of this novel two-part craniotomy technique, including diploic bone drilling, permitted safe access to the midline and the tumor without injury to the superior sagittal sinus (SSS), as illustrated in Figure 11.

The dura was then opened to the lateral wall of the patent SSS anteriorly and posteriorly after separating the convexity dura from the top of the tumor. Once clear access to the tumor was available, the tumor was removed from the lateral wall of the SSS, and the involved falx was excised. A Simpson grade 1 removal was accomplished.

The dura was then reconstructed with a pericranial graft. The bone pieces were connected one to another on the side of the inner table with titanium plates and screws. The bone flap was repositioned and secured to the surrounding bone with recessing outer table screws and the use of hydroxyapatite cement. Skin closure was performed, and the patient was uneventfully extubated.

Postoperative MR imaging confirmed the gross total removal of the meningioma. Her postoperative course was uneventful, and the patient was doing well at the last follow-up.

## Discussion

There exist several methods for crossing the midline and the superior sagittal sinus (SSS) when performing a vertex craniotomy [3-5]. This technical report demonstrates the use of the “two-part parasagittal” craniotomy for this purpose. Institutionally, this technique has had satisfactory results in all regions of the SSS and torcula.

When the surgeon is confronted with an irregular inner table related to tumor-associated hyperostosis, the area of involvement is limited, and when not involving the dura over the SSS, bone flap removal is not a problem. However, when the irregularity of the inner table is more widespread, as with HFI, or is close to the midline, then the risk of a dural tear and injury to parasagittal veins or the SSS is higher [1,2]. Using a two-part bone flap allows the exposure of the inner table lateral to the midline, so that safe dissection of the epidural space under direct vision can be accomplished. When there is a significant width of an irregular inner table, then drilling out the diploic bone permits the creation of a thin inner table that can be removed piecemeal with a thin footplate, a small diameter rongeur, such as a 2-mm Kerrison rongeur. Since the outer table is untouched, no reconstruction is warranted during closure to preserve cosmesis.

Falcine and parasagittal meningiomas present key SSS-related technical considerations, as highlighted in this article. The SSS is a challenging structure to operate around, given the difficulty in controlling an inadvertent laceration [6]. Oberman et al. recently reported that the SSS may be displaced by as far as 16.3 mm in the majority of individuals [7]. There exists limited literature on surgical approaches to extra-axial pathology around the SSS, with most reports being concerned with the treatment of epidural hematoma. Numerous case reports detail treatment strategies that entail both avoidance and craniectomy of the midline diastatic bone comprising the sagittal suture [4,5,8-12]; typically, avoidance of midline craniectomy is based on concern for a direct injury of the underlying SSS. However, when it is necessary to expose the SSS to facilitate improved extra-axial dissection, the method described here offers a safe and efficacious means of removing the frontal bone, even when thickened. In another surgical series of calvarial masses overlying the dural sinuses, the removal of bone overlying the SSS was found to have an insignificant increase in morbidity for select patients [13].

The described technique has been used in several cases over the years by the senior author, providing a solution for the problem of the irregular inner table and the need to dissect the dura over the midline.

## Conclusions

Diploic bone channel drilling is a technique that can be used to create a thin lip of the inner table that can be removed piecemeal for safe dissection of the dura crossing the midline in a two-part parasagittal craniotomy. This technique may be utilized in a safe, consistent, and efficacious manner to avoid injury to the superior sagittal sinus when access around the same is warranted, for instance, in cases of falcine and parasagittal tumors.
